# Carbon-coated iron nanopowder as a sintering aid for water-atomized iron powder

**DOI:** 10.1038/s41598-022-22336-4

**Published:** 2022-10-25

**Authors:** Swathi K. Manchili, F. Liu, E. Hryha, L. Nyborg

**Affiliations:** 1 Department of Industrial and Materials Science, Chalmers University of Technology, 41258 Gothenburg, Sweden; 2 SIMaP, Grenoble INP, CNRS, Université Grenoble Alpes, Grenoble, France

**Keywords:** Structural materials, Nanoscale materials, Nanoparticles, Engineering

## Abstract

The paper examines the influence of carbon coating on iron nanopowder used as a sintering aid for water-atomized iron powder. Iron nanopowder without such a coating was used as a reference sintering aid to isolate the influence of the carbon coating. Both nanopowder variants were characterised using XPS and HRTEM. The results showed a core–shell structure for both variants. The iron nanopowder is covered by a 3–4 nm thick iron oxide layer, while the carbon-coated iron nanopowder is encapsulated with several nanometric carbon layers. Thermogravimetry conducted in a pure hydrogen environment shows a multipeak behaviour for the carbon-coated iron nanopowder, while a single peak behaviour is observed for the iron nanopowder. Two types of micro/nanobimodal powders were obtained by mixing the nanopowder with water-atomized iron powder. Improved linear shrinkage was observed during sintering when the carbon-coated iron nanopowder was added. This can be explained by the reduction in surface diffusion in the nanopowder caused by the carbon coating, which allows the nanopowder to sinter at higher temperatures and improves densification. Carbon and oxygen analysis, density measurements, optical microscopy and JMatPro calculations were also performed.

## Introduction

Press and sinter is a powder metallurgical (PM) manufacturing route in which shaping techniques such as uniaxial compaction are used to process metal powder to the required shape, after which the compact material is sintered to make it useful for application. During sintering, the part is heated so that the metal particles bond to one another, which imparts the required strength. The strength obtained is proportional to the density of the component^1^. Hence, it is essential to improve density to improve the properties of PM components and subsequently widen their range of applications. Density can be improved in many ways, for example by adding a sintering aid. Nanopowder is one such sintering aid and is known to lower the activation energy required for sintering^2,3^. The addition of nanopowder has been explored in the field of metal injection moulding (MIM), where enhanced properties were observed^4^.

Nanoparticles have unique size-dependent properties, which are attributed to the large fraction of atoms present at the surface of these materials in comparison to their counterbulk materials^5,6^. These unique properties have been exploited for applications in areas such as chemical analysis, microelectronics, biological sensors and other functional applications^7,8^. However, for nanoparticles to be useful in these applications, it is important that they remain stable and retain their size. Because of their large surface-to-volume ratio, there is excess surface energy associated with them. Therefore, they have a strong tendency to coalesce, which leads to significant changes in processability. Nanopowder can be coated with carbon, which stabilizes the nanoparticles against agglomeration and coalescence. Carbon-coated iron nanopowder has been used in applications such as magnetic data storage, magnetic toners in xerography, contrast agents in magnetic resonance imaging, catalyst supports and drug and gene delivery systems^9–13^. Additionally, the carbon-coating provides a source of carbon, which would otherwise need to be added separately to set the indented final composition of the sintered steel^14^.

In previous work, the authors explored the addition of pure iron nanopowder as a sintering aid to water-atomized iron powder^15^. The sinter curves revealed a pronounced influence of the nanopowder addition on the sintering behaviour of these micro/nano bimodal powder compacts. Sintering experiments conducted at intermittent temperatures to track the development of sinter necks with the increase in temperature and subsequent fractographic analysis of the compacts revealed that the sintering of nanopowder at temperatures as low as 600 °C, below the onset of the sintering of the micrometre-sized base powder. Though, the linear shrinkage of water-atomized iron powder improved with the addition of iron nanopowder as a sintering aid, a further improvement is needed. This is to enable adaptation of near full density press and sinter PM parts in areas where these are not currently in use, where improved performance is needed. To achieve near full density, hot isostatic pressing (HIP) is employed. In order to enable capsule-free HIP, closed porosity (95% theoretical density) or near closed porosity is needed. This research is part of a larger framework to measure the efficacy of using nanopowder as a sintering aid to achieve closed porosity in water-atomized iron powder.

This study explores the possibility of employing carbon-coated iron nanopowder instead of pure iron nanopowder as a sintering aid. The study’s primary objective is to determine how the presence of carbon on the surface of the iron nanopowder affects the sintering of water-atomized iron powder.In addition, the carbon coating on the iron nanopowder would serve as a source of carbon and participate in the reduction of oxides. In the study, two systems are selected: carbon-coated iron nanopowder and iron nanopowder without any coating. The base powder in both cases is micrometre-sized water-atomized iron powder. X-ray photoelectron spectroscopy and high-resolution transmission electron microscopy are used to characterize both kinds of nanopowder in detail. The sinterability of the two variants of micro/nano bimodal powder compacts is compared. The focus of the study is on how the presence of a carbon-coated iron nanopowder affects the sintering of water-atomized iron powder.

## Results and discussion

### XPS of nanopowder

**Figure 1:**
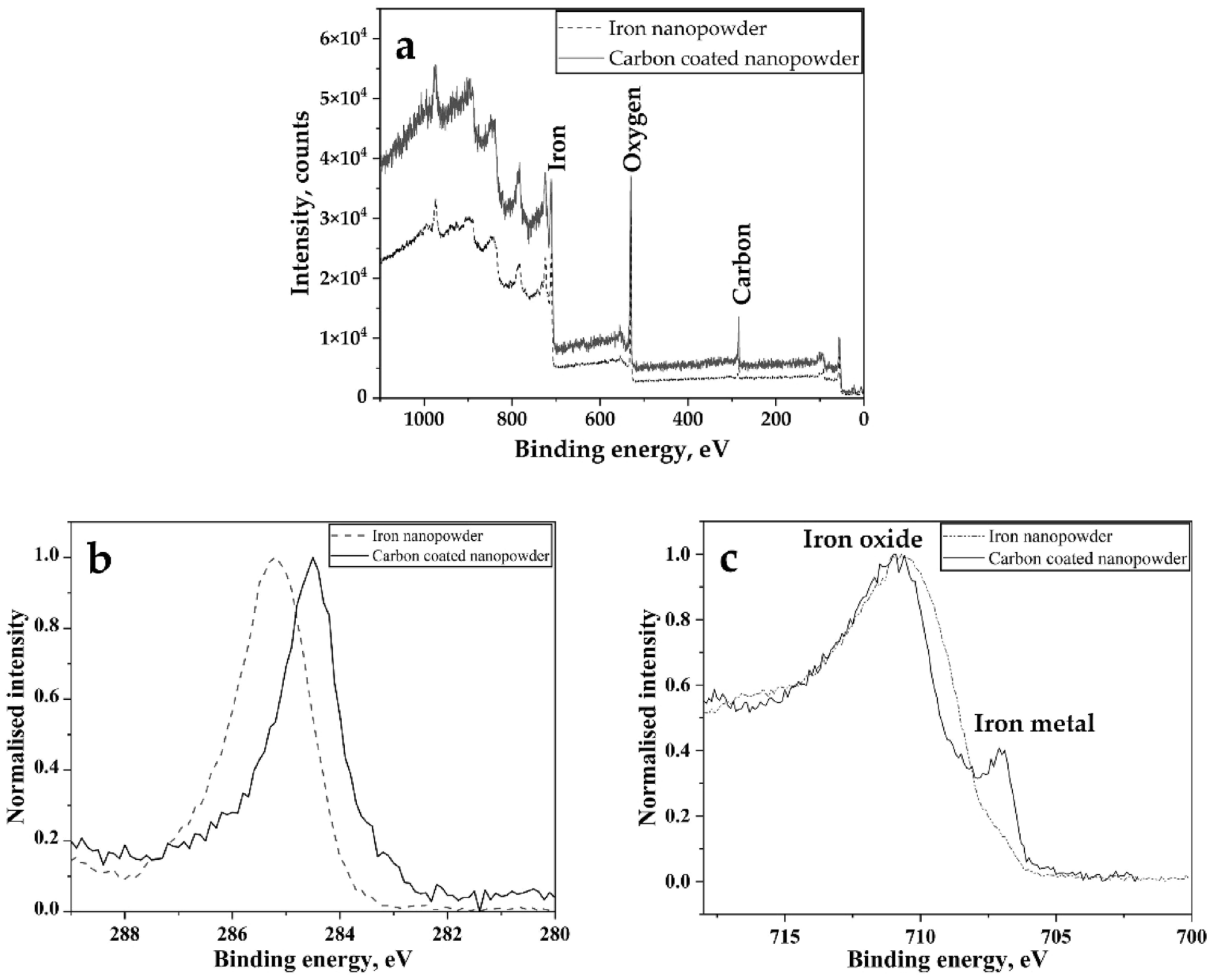
(**a**) XPS spectra recorded from the as-received surfaces of both nanopowder variants showing iron, oxygen and carbon peaks in the survey scan, (**b**) high-resolution XPS spectra for carbon (C1 s) from both variants showing the change in C1 s peak positions, and (**c**) high-resolution XPS spectra for iron (Fe2p^3/2^) showing the presence of both oxide and metal iron peaks in both cases but with a higher relative intensity of metallic iron for the carbon-coated nanopowder.

X-ray photoelectron spectroscopy (XPS) measurements were obtained to investigate the surface chemical characteristics of both iron nanopowder (Fe NPs) and carbon-coated iron nanopowder (CC NPs). Figure 1 presents the survey spectra of both Fe NPs and CC NPs as well as the high-resolution spectra for carbon (C1 s) and iron (Fe2p^3^/^2^). The survey scan of both nanopowders with binding energies from 0 to 1100 eV that was conducted on the as-prepared sample surface shows the elements present in the near surface region of the powder, as illustrated in Fig. 1a. The spectra reflect the presence of iron, oxygen and carbon indicated by their characteristic peaks.

The high-resolution XPS results clarify the surface characteristics of the nanopowder variants. Figure 1b shows the different peak positions for carbon (C1 s) for the two nanopowder variants. The C1 s peak position for the carbon-coated nanopowder is 284.4 eV, while it is 285.2 eV for the iron nanopowder. These positions correspond to graphitic carbon and carbonaceous contamination layers on the two kinds of nanopowders, respectively^16,17^. The high-resolution XPS spectra of iron (Fe2p^3/2^ region) in Fig. 1c show strong iron oxide peaks but different relative intensities for metallic iron. The highest relative amount of metallic iron is indicated for the carbon-coated nanopowder. Based on, from the relative intensities of the iron oxide and metallic iron peaks, the overall oxide thickness can be depicted. This is because XPS is sensitive to the outer 3 to 5 nm of a sample, as the signal comes from a maximum of three times the attenuation length of the photoelectrons^18^. In a previous study, a detailed assessment of the oxide layer thickness was carried out involving the application of different models/approaches, and it was found that the thickness of the oxide scale on Fe NPs is approximately 3 nm^19^. Here, to provide further insight into the difference in the surface chemical structure of the two variants, the XPS results are complemented with high resolution transmission electron microscopy.

### High resolution transmission electron microscopy (HRTEM)

**Figure 2:**
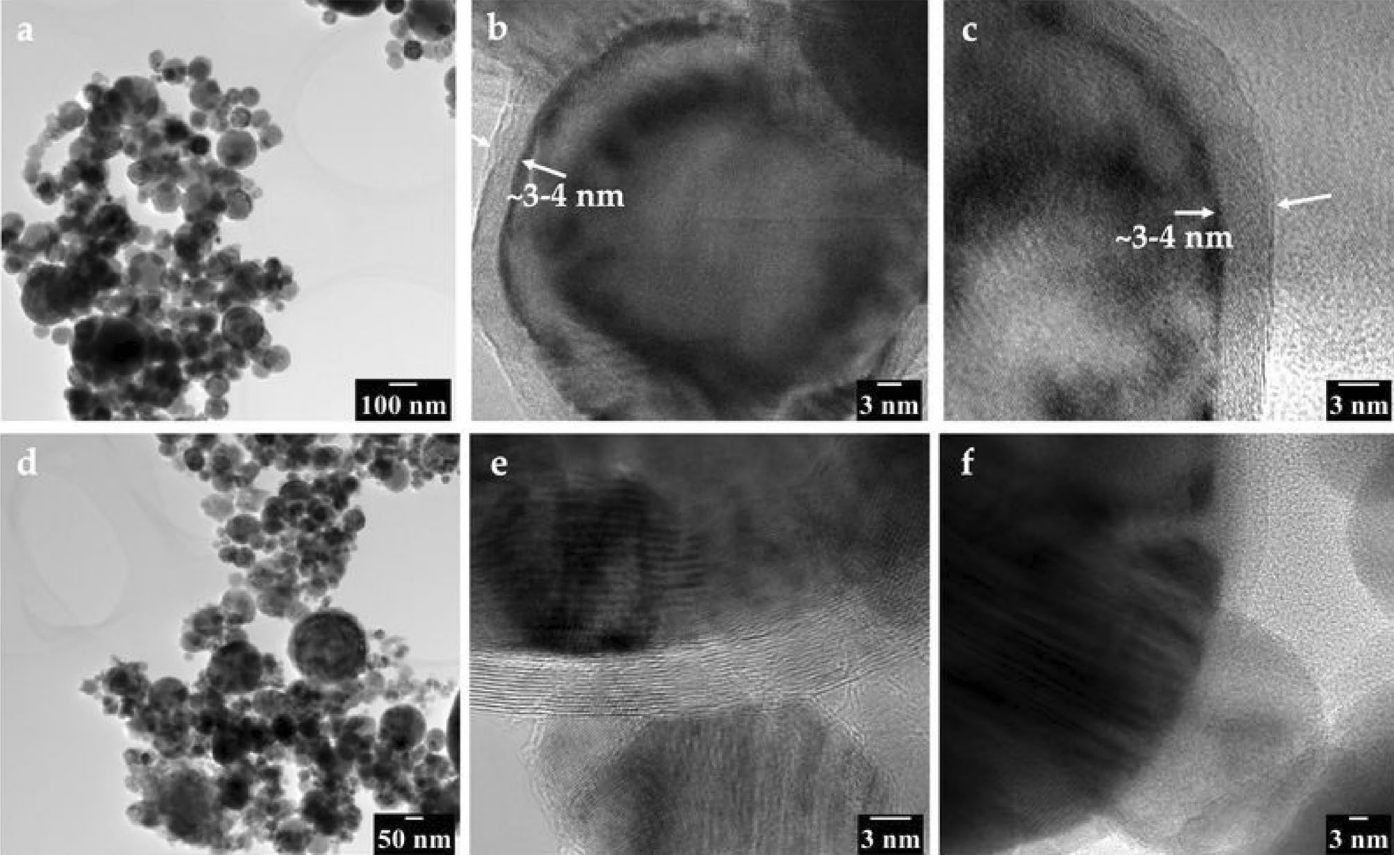
(**a**) TEM images of iron nanopowder (Fe NP) showing the size and morphology of the particles, (**b**, **c**) HR TEM images revealing the thickness of the surface oxide present on the Fe NP, (**d**) TEM images of carbon-coated iron nanopowder (CC NP) showing size and morphology of the particles and, (**e**, **f**) HR TEM images illustrating the core–shell structure with the iron core tightly coated with graphitic carbon layers.

Figure 2 presents the TEM images of both nanopowders, revealing their shape, size and surface structure characteristics. Figure 2a shows the TEM image of the Fe NPs at low magnification illustrating several particles. The particles are less than 100 nm in size. The nanopowder is observed to be spherical. The high-resolution micrographs in Fig. 2b, c show that the particle fits the core–shell model and that the oxide shell is 3–4 nm thick. This complements the authors’ previous XPS studies conducted on the same powder^19^.

Figure 2d depicts several particles of CC NPs at low magnification. Similar to the Fe NPs, the CC NPs were under 100 nm in size and had a spherical morphology. The HR TEM technique was used to investigate the structure of the carbon layer surrounding the nanopowder. The results are shown in Fig. 2e, f. The presence of a multilayer carbon coating, which is compact and adherent on the iron particles, can be observed. The iron core is entirely coated by a multilayer carbon shell, which is an indication of an integrated core–shell structure. The interplanar spacing was measured to be approximately 0.30–0.35 nm, which is similar to the value for graphite^13^. The HR TEM images show clear difference in structure between iron oxide and graphitic layers on the surface: CC NP always shows the characteristic layered structure of graphitic carbon under HR TEM. This morphology is so characteristic for carbon nanotubes that it is routinely used to count the number of layers in the carbon nanotube. In order to make this point clear, image in its original size is given in the supplementary information (Fig. S1). On the other hand, the oxide layer on the Fe NP shows more complex lattice structure in HR TEM images (Fig. S2). The image was recorded along the [1-11] zone axis of the iron core. The distance and angles of the bright spots which represent the colums of atom were measured to be 1.975 Å and 60°, respectively which matches well for iron. Additionally, the extracted Fourier transform pattern (from the oxide region) was indexed as [1-21-3], zone axis of Fe_2_O_3_. The apparent tight covering of the nanoparticles by the carbon shell indicates that both the iron oxide and metallic iron peaks detected by XPS for this powder must come from the core below and that their relative intensities reflect the difference in damping below the carbon shell. Additionally, it can be stated that the carbon shell imposes much less dampening of the XPS signals from the core than an oxide layer, and the actual oxide layer (presumably located as an interface compound) must be much thinner than in the case of the iron nanopowder.

### Thermal analysis

**Figure 3:**
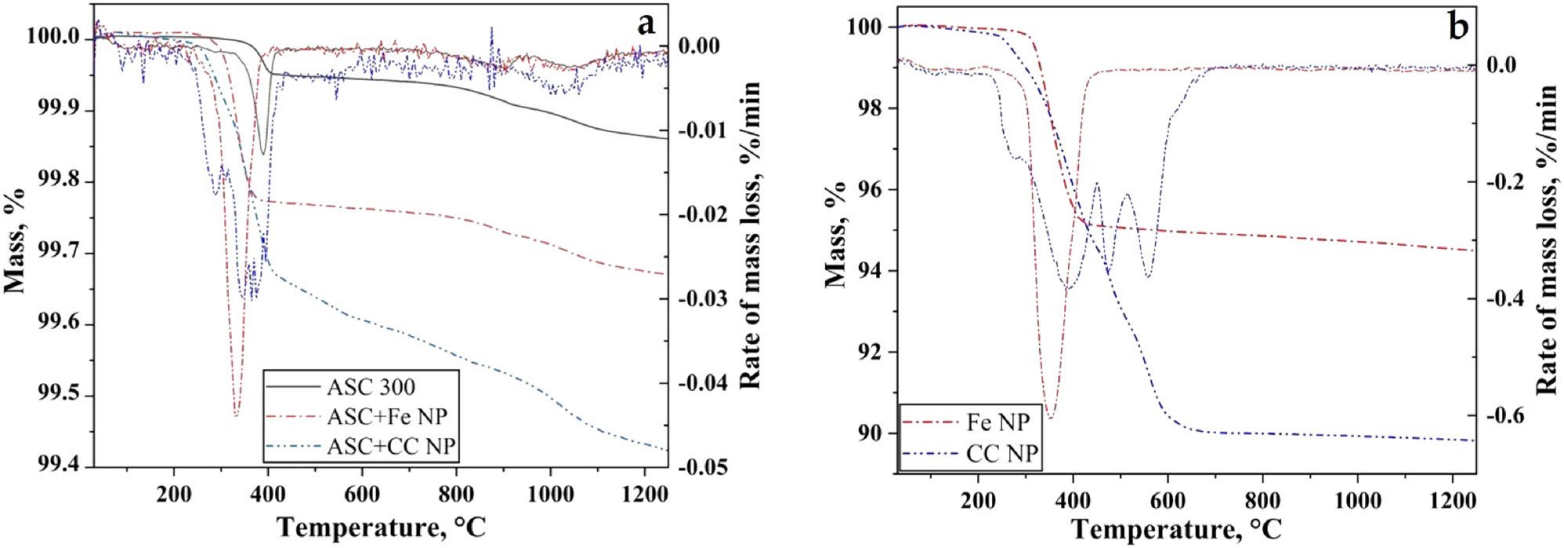
Thermogravimetry plots depicting the change in mass and rate of mass change for (**a**) micrometre-sized powder with and without nanopowder additions and (**b**) iron nanopowder and carbon-coated iron nanopowder alone.

Figure 3a presents the thermogravimetric graphs for the micrometre-sized reference powder (ASC 300) and the micro/nano bimodal powder with the two different variants of nanopowder. The change in mass is recorded as a function of temperature in pure hydrogen. The addition of nanopowder resulted in a much greater mass loss. Figure 3b depicts the change in mass of the Fe NPs and CC NPs and the mass loss rate as a function of temperature under the same conditions. The total mass loss is 5.6% and 10% for the Fe NPs and CC NPs, respectively. In the case of the Fe NPs, the mass loss can be attributed to oxygen removal through the reduction of surface oxides in a single step. The surface oxide (Fig. 2) is reduced at temperatures below 500 °C, above which the change in mass is negligible. The rate of change in mass, which is the first derivative, is depicted in Fig. 3b and shows a single peak at 355 °C, which is the temperature of the maximum reduction rate. The mass change is observed over a narrow range of temperatures. An existing detailed study of the reduction behaviour and kinetics of Fe NPs^20^ clearly points to the existence of a single reduction step. The CC NPs show a mass loss of approximately 10%, and this mass loss occurs over a temperature range of between 300 and 700 °C. Although the change in mass loss appears to be a single step, the derivative shows at least four peaks at 275, 383, 475 and 558 °C. The mass loss is hence seemingly attributable to both reduction by hydrogen at lower temperatures and to the action of carbon. Figure 3a illustrates that the total mass losses observed were 0.15, 0.34 and 0.59% for the ASC 300, ASC + Fe NP and ASC + CC NP powders, respectively. The difference in total mass loss is attributable to oxygen removal from the nanopowder. The mass loss was higher for the ASC + CC NPs than the ASC + Fe NPs. A total mass loss of 0.34% was observed for the ASC + Fe NPs, while the ASC + CC NPs experienced a mass loss of 0.59%. For ease of understanding, the TG curve can be divided into two different regions. Region one corresponds to the temperature range below 500 °C, where the mass loss is related to the surface oxide reduction for both the nanopowder variants and ASC 300. Above this temperature, there is the second temperature range, during which the reduction of more stable particulate oxides predominantly found on the base powder and potential internal oxides occurs. The reduction of particulate oxides at higher temperatures is examinedin detail in other studies^21,22^. Nevertheless, the major fraction of the total mass loss occurs in the first region. The increase in the mass loss between ASC 300 and the micro/nano bimodal powder is hence attributed to the reduction of surface oxide present in the nanopowder. The increase in mass loss in the case of the CC NPs is associated with the loss of both oxygen and carbon, where the latter is consumed for the carbothermal reduction of oxides at elevated temperatures. Hence, the addition of carbon via the CC NPs will also affect the reduction of the particulate oxides, as this reduction will not occur to the same extent when sintering ASC 300 alone in the present case, because no graphite is added.

### Carbon and oxygen analysis

Table 1 shows the carbon and oxygen levels for the different powder variants and mixes and their sintered conditions. The carbon content of the ASC 300 powder was 0.005 wt.%, whereas that of the Fe NPs was less than 0.1 wt.%; therefore, the total carbon content in the ASC + Fe NP mix should be a maximum of 0.006 wt.%. When sintered under a pure hydrogen environment, the carbon content is reduced to a negligible 0.002 wt.%. Consequently, approximately two thirds of the carbon is utilized during sintering, as carbon is the predominant reducing agent at elevated temperatures^23^. Hence, it is suggested that this carbon loss results from the reduction of particulate oxides at higher temperatures.

**Table 1:** Carbon and oxygen values before and after sintering.

Material	Carbon, wt.%	Oxygen, wt.%
Fe NP, powder	0.06 ± 0.0007	5.5 ± 0.033
CC NP, powder	4.7 ± 0.023	7.5 ± 0.027
ASC 300 + Fe NP, powder	0.006 ± 0.0002	0.28 ± 0.002
ASC 300 + CC NP, powder	0.24 ± 0.003	0.37 ± 0.002
ASC 300 + Fe NP, sintered	0.002 ± 0.0002	0.02 ± 0.001
ASC 300 + CC NP, sintered	0.014 ± 0.0003	0.013 ± 0.0007

In the ASC + Fe NP powder, the surface oxide present on the Fe NPs contributes most of the oxygen content. When Fe NPs were added to ASC 300, the oxygen content increased from 0.1 to 0.28 wt.%, which is an approximately threefold increase. The 3 nm-thick surface oxide on the Fe NPs amounts to 5.5 wt.% oxygen content in the Fe NPs. Upon sintering, the oxygen content was reduced to 0.02 wt.% in the ASC + Fe NP compact, which accounted for only 7% of the total oxygen content. The remaining 93%, present to a large extent in the form of iron oxide on the surface of the Fe NPs and in the form of iron-rich surface oxides and possibly some particulate oxides in ASC 300, was reduced during the course of sintering.

For the CC NPs, the carbon content was found to be 4.7 wt.%. When added to ASC 300, the total carbon content should be supposed to be 0.24 wt%, which is two orders of magnitude higher than the carbon content of the ASC + Fe NPs. When sintered, the carbon content was reduced to 0.01 wt.%. Thus, only 6% of the total carbon remained after sintering. The remaining 94% was utilized in the reduction of oxides. The oxygen content in the CC NP was 7.5 wt.%. When mixed with ASC 300, the powder mix has 0.37 wt.% oxygen. Upon sintering, 97% of the total oxygen content was reduced, as only 0.013 wt.% remained. Consequently, carbon added via the nanopowder is highly active and directly involved in surface oxide reduction. Furthermore, it supposedly contributes to the overall reduction of all surface oxides in the material, hence the significant carbon loss and extremely efficient reduction. The final level of oxygen of approximately 0.01 wt% is well below what is observed in conventional press and sinter material sintered at 1250 °C.

### Sintering

**Figure 4:**
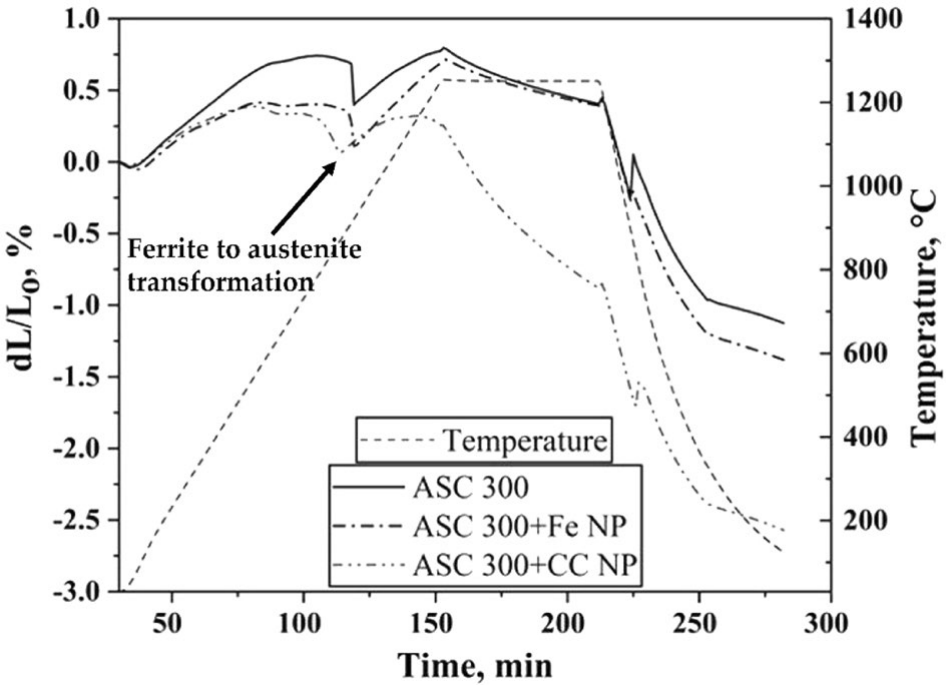
Sintering curves of ASC 300, ASC + Fe NPs and ASC + CC NPs compacts at 1250 °C in pure hydrogen at a heating rate of 10 °C/min and a cooling rate of 30 °C/min.

Compacts of ASC 300 and ASC 300 mixed with the different variants of nanopowder at a ratio of 95:5 were subjected to sintering runs in a dilatometer (DIL). In addition to the sintering shrinkage, the materials undergo dimensional changes associated with thermal expansion, allotropic transformations and events that lead to the development of their microstructure during heating and cooling. Figure 4 presents the sintering curves for the compacts sintered at 1250 °C when heated at 10 °C/min, isothermally held for 60 min and cooled at 30 °C/min. It should be noted that all three compacts were sintered under identical sintering conditions. During the runs in DIL, the change in the linear dimension is measured as a function of time and temperature. The curve can be divided into three different stages: heating, isothermal holding and cooling. During the heating stage, the material is expected to expand. Sintering occurs place during the heating stage, which can be seen as a deviation from the expected expansion. During isothermal holding, the compact undergoes shrinkage undergoes shrinkage, the extent of which depends on the sinterability of the material. Then, during the cooling stage, the compactness is expected to shrink. Dimensional changes are also subjected to phase transformations. In this case, similar to typical iron systems, the transformation from body-centred cubic (BCC) ferrite to face-centred cubic (FCC) ferrite occurs during the heating stage.

The linear shrinkage values are 1, 1.3 and 2.6% for the ASC 300, ASC + Fe NP and ASC + CC NP sintered compacts, respectively. There is hence a clear difference between the sintering behaviours of the compacts with and without nanopowder. This difference, observed in the low temperature regime, is discussed in detail by the authors elsewhere^15^. The change in the slope of the sinter curves during both heating and cooling at approximately 900 °C for the ASC and ASC + Fe NP compacts and at lower temperatures for the ASC + CC NP compact can be explained by the phase transformations that occur. For the ASC 300 and ASC + Fe NP compacts, the BCC to FCC transformation occurred at ~ 912 °C, as expected for pure iron. It should be noted that the carbon content in ASC 300 and the ASC + Fe NP powder was 0.005 and 0.006 wt.%, respectively.

Improved linear shrinkage was observed for the ASC + CC NP compact compared to the ASC + Fe NP compact. The HRTEM results show a coating of graphitic carbon over the entire iron particle. Research has shown that the carbon present on the surface of nanoparticles serves as a barrier to the sintering of these particles^6^. The surface diffusivity of silver in the presence of carbon coating, for example, has been assessed using in-situ HRTEM techniques and found so be several orders of magnitude lower than the values obtained from bulk silver at high temperatures and when extrapolated to room temperature. Based on the experiments in ref.^2^, it was suggested that when the surface of silver nanoparticles is carbon-coated, the diffusion of atoms from the surface to the neck occurs through the carbon layer. As the neck grows, the carbon coating is pushed outwards from the neck region to drive further growth. This process is slower than the surface diffusion of pure silver, which results in lower initial diffusivity values in the presence of carbon. In the present case, a thin iron oxide layer is sandwiched between the carbon coating and the iron core of the CC NPs, as shown by the XPS and HRTEM analyses. During the heating cycle, the iron oxide is expected to be reduced for the sintering of the nanopowder to proceed in the lower temperature regime for the nanopowder. However, in the case of the CC NPs, the carbon coating is expected to hinder the reduction process by hydrogen and to lower the surface diffusivity of the iron. This would lead to reduced sintering of the nanopowder in the low-temperature regime, contrary to what is expected in nanopowder-added sintering. The sintering is then activated once the carbothermal reduction of the sandwiched oxide becomes possible as the temperature increases. Consequently, hydrogen is tentatively not as active to reduce this sandwiched oxide layer of the CC NPs as long as the carbon layer is intact.

### Density

**Figure 5:**
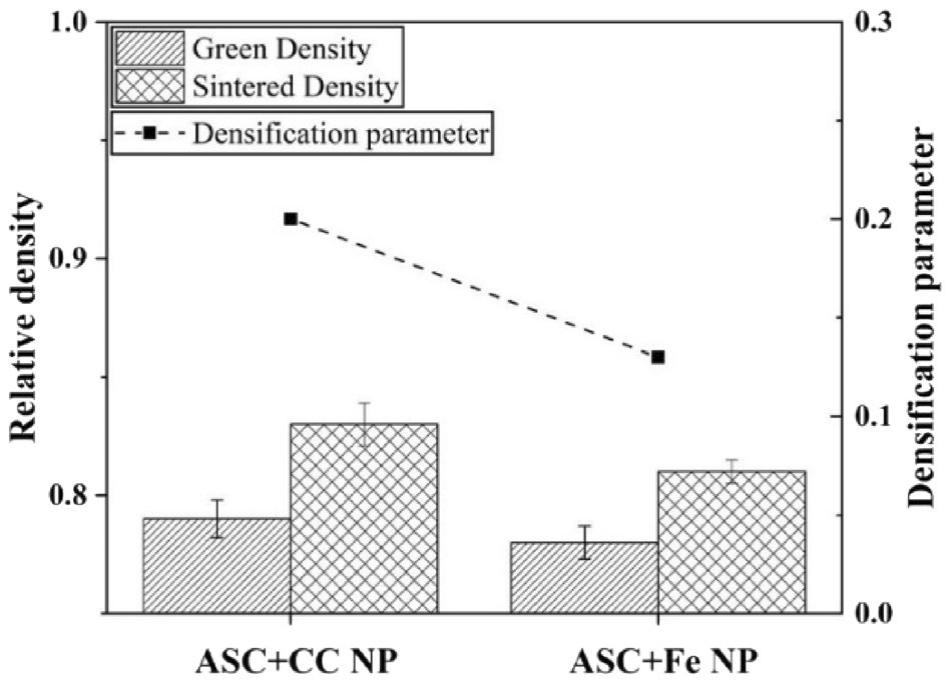
Green and sintered densities in relative terms of the ASC + CC NP and ASC + Fe NP compacts along with densification parameters.

A comparison between the green and sintered density data, shown in Fig. 5, for both compacts with added nanopowder indicates that the ASC + CC NPs had a slightly higher green density than the ASC + Fe NP compact. The relative density for the ASC + Fe NP green compact was 0.78, whereas for the ASC + CC NP green compact, it was 0.79. The sintered density followed the same trend as the green density; namely, the compact ASC + CC NPs had a higher final sintered density than the ASC + Fe NPs. The relative densities for the final sintered compacts were 0.81 and 0.83 for ASC + Fe NP and ASC + CC NP, respectively. Since the green densities of the two compacts differed slightly, the densification parameter can be used to evaluate the effect of each nanopowder on sintering. The densification parameter is defined as the change in density during sintering divided by the change needed to attain a pore-free solid^2^. It is given by the following equation:

1 $$\psi = \frac{{\rho_{s} - \rho_{g} }}{{1 - \rho_{g} }}$$

where *ρ*_*s*_ is the sintered density and *ρ*_*g*_ is the green density. The theoretical density of iron is now considered to be 7.9 g/cc. The densification parameter is then found to be 0.2 for ASC + CC NP and 0.13 for ASC + Fe NP sintered compacts. Thus, the densification of the compacts containing CC NPs was greater than that of the compacts containing Fe NPs. Even if closed porosity is not reached, the higher densification parameter of the CC NP compact indicates that there could be a potential effect of shifting the densification towards more closed porosity taking advantage of, in particular, the effect of carbon coating.

### Microstructure of sintered compacts

**Figure 6:**
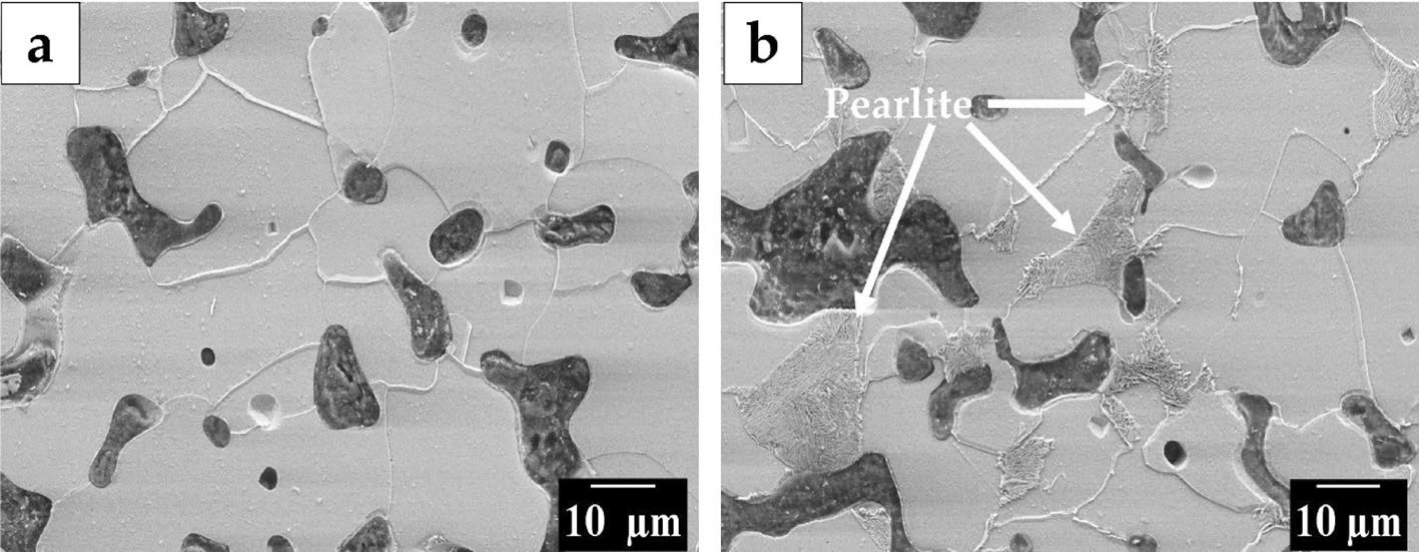
SEM micrographs of sintered compacts showing the microstructure of Sintered compacts of (**a**) ASC + Fe NP, and (**b**) ASC + CC NP.

Figure 6 presents the microstructure of the ASC + Fe NP and ASC + CC NP sintered compacts. Figure 6a reveals ferrite grains in the ASC + Fe NP sintered compact, while Fig. 6b shows a combination of ferrite and pearlite in the ASC + CC NP sintered compact. The total volume fraction of pearlite is lower, and Fig. 6b shows the region in which pearlite is present. Using the Trainable Weka Segmentation tool, which is part of the Fiji ImageJ freeware, the volume fraction of pearlite was estimated to be 2%. The percentage of carbon needed for 2% pearlite, according to JMatPro calculations, is 0.05 wt.%. Based on a chemical analysis, it was established that the total carbon content after sintering of the ASC + CC NP compact was 0.014 wt.%. Pearlite pockets, hence seem to form in the regions where carbon remains after sintering are present. It should be noted that the carbon is not uniformly distributed throughout the compact and is expected to occur where nanopowder would have been present during the sintering cycle. Clearly, it is important that carbon loss is accounted for when selecting the amount of sintering aid.

### Hardness of sintered compacts

**Figure 7:**
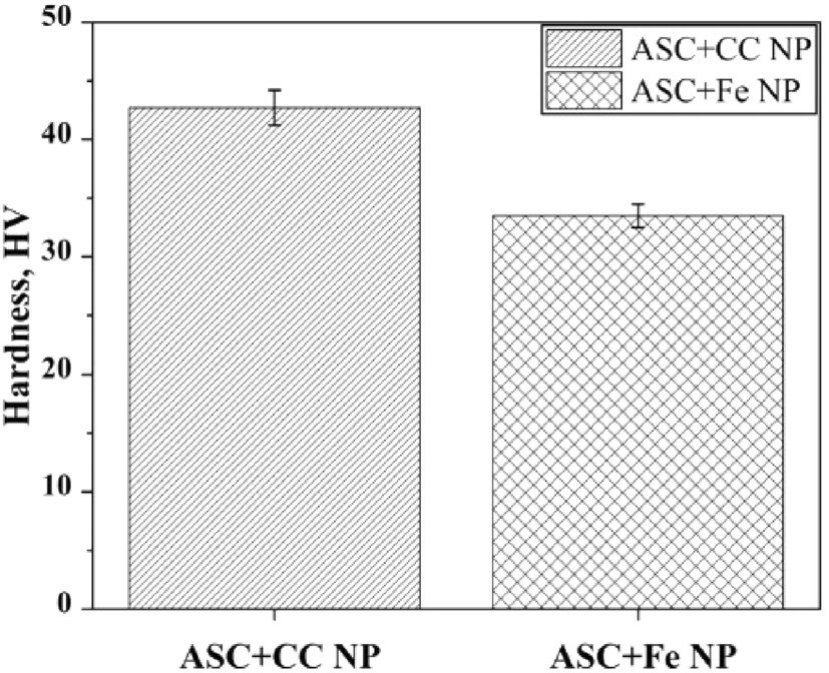
Plot showing the Vickers hardness of the ASC + Fe NP and ASC + CC NP sintered compacts.

Figure 7 shows the apparent hardness measured at 1 kg for the sintered compacts of ASC + Fe NPs and ASC + CC NPs. The Vickers hardness value of the ASC + Fe NP sintered compact was 33 HV, in line with the expected value for pure iron, while that of the ASC + CC NP sintered compact was 42 HV due to the finite amount of pearlite.

The use of carbon-coated iron nanopowder versus uncoated nanopowder as a sintering aid for water-atomized iron powder was explored. Both nanopowder variants revealed a core–shell structure, where the iron nanopowder consisted of an iron core and oxide shell with a thickness of 3–4 nm, while the carbon-coated iron nanopowder had a graphitic carbon shell of similar total thickness with a potentially thinner iron oxide layer sandwiched between the carbon shell and the iron core. Thermogravimetry and chemical analysis were used to investigate the behaviour of the nanopowder with respect to oxide reduction and sintering and the effect of adding nanopowder of either variant to micrometre-sized base powder. Generally, adding nanopowder to micrometre-sized powder significantly reduces the powder compressibility. However, the shift from iron nanopowder to carbon-coated powder yielded marginally improved compressibility. Improved linear shrinkage of compacts during sintering was observed with the addition of both kinds of nanopowder to the micrometre-sized powder and particularly with the addition of the carbon-coated variant. The carbon coating may inhibit the sintering enhancement by nanopowder at lower temperatures, while at higher temperatures as the carbon partakes in the oxide reduction reactions. When carbon-coated nanopowder was added to the micrometre-sized base powder at a ratio of 5:95, an initial total carbon level of 0.24% was achieved. However, over 90% of this total carbon is lost during sintering. The remaining carbon results in the local formation of pearlite islands, which is consistent with the slightly higher hardness for the sintered compacts with carbon-coated nanopowder compared to the sintered compacts containing iron nanopowder. In conclusion, it can be stated that the carbon-coated iron nanopowder addition, compared to the iron nanopowder addition, yielded a greater improvement of both the green and sintered density of water-atomized iron powder.

## Materials and methods

### Materials

Pure iron nanopowder (Fe NP) (Product number: 746851) and carbon-coated nanopowder (CC NP) (Product number: 746827) were procured from Sigma–Aldrich. The particle size of both grades of nanopowder was below 100 nm. The particle size distribution of nanopowder variants is given in supplementary information. A micrometre-sized powder of pure iron, henceforth referred to as ASC 300, with a D_50_ of 30 µm was supplied by Höganäs AB. The micrometre-sized powder and nanopowder were combined at a ratio of 95–5 wt.%, yielding two variants of micro/nanobimodal powder, ASC 300 + 5 wt.% Fe NPs and ASC 300 + 5 wt.% CC NPs. Mixing was conducted in a tumbler placed in a glove box for a few hours. Storage and handling of the nanopowder was performed in a nitrogen-filled glovebox prior to any processing, sample preparation or characterisation.

### X-ray photoelectron spectroscopy

Both nanopowder variants were subjected to X-ray photoelectron spectroscopy (XPS) using a PHI Versaprobe III equipped with a monochromatic Al K_α_ (1486.6 eV) X-ray source. Ultrahigh vacuum conditions of 10^−9^ mbar were maintained during the powder analysis. The sample surface and the X-ray beam were placed perpendicular to one another with a take-off angle is 45° with respect to the sample surface. Pass energies of 140 and 26 eV were used for the survey scans and for high-resolution scans, respectively. Before measuring commences, energy calibrations were conducted using pure elemental standards of gold, silver and copper. A graphite standard sample was used for the exact peak position. The binding energies were referenced to the graphitic sp_2_ hybridized carbon for C1 s set at 284.4 eV^16,24^. The samples for the XPS analysis were prepared inside the glove box. Loose powder was pressed between aluminium plates, and the plate was mounted on the sample holder. The XPS data were analysed using the MultiPak V9.0 software supplied with the instrument.

### Transmission electron microscopy

Transmission electron microscopy (TEM) observations of the nanopowder variants were performed via high-resolution transmission electron microscopy (HRTEM) using an FEI Titan 80-300 operated at 200 kV. The nanopowder was first dispersed in isopropanol and placed in an ultrasonic bath for 15 min to reduce particle agglomeration. A small droplet of the nanopowder dispersed in this solution was then deposited onto a holey carbon copper grid using a pipette. The copper grid containing the nanopowder was placed in a TEM sample holder and loaded into the TEM instrument. Bright-field TEM (BF-TEM) was used to obtain information about the nanopowder size and shape and to determine its structure and morphology.

### Thermal analysis

Thermogravimetric (TG) analysis of the nanopowder and micro/nano bimodal powder samples was performed using simultaneous thermal analyser STA 449 F1 Jupiter equipment (Simultaneous Thermal Analyser, Netzsch Thermal Analysis GmbH, Germany). Powder of the required mass (500 mg for nanopowder and 2 g for bimodal powder) was loaded to an alumina crucible in the glove box. In the TG equipment, the samples were heated to 1350 °C at a heating rate of 10 °C/min, and the change in mass was recorded as a function of temperature. High purity hydrogen gas (99.9999%) was used with the aim of reducing the surface oxide early on during heating. A flow rate of 100 ml/min was maintained throughout the process.

### Carbon and oxygen analysis

The bulk carbon and oxygen levels of both the powder and sintered compacts were determined using LECO TC-600 and LECO CS-844 instruments. The oxygen present in the sample reacted with the crucible, forming CO and CO_2_. The amounts of CO and CO_2_ were measured using infrared (IR) sensors and employed to estimate the amount of oxygen. To measure carbon, the sample was combusted in an induction furnace under the flow of oxygen. The amount of carbon in the sample was determined based on the CO and CO_2_ formed via reactions between the carbon in the sample and oxygen.

### Compaction and sintering

Both variants of micro/nano bimodal powder were uniaxially compacted to cylindrical disks of 10 mm in diameter and 4 mm in height. No lubricant was added for compaction. Sintering was performed using a DIL 402C horizontal push rod dilatometer (Netzsch Thermal Analysis GmbH, Germany: DIL) under high purity hydrogen gas (99.9999%). A sintering temperature of 1250 °C was employed at a heating rate of 10 °C/min, followed by isothermal holding for 60 min at peak temperature and cooling to room temperature at a rate of 30 °C/min. The maximum final temperature of 1250 °C is the same temperature used to sinter ferrous powder in industrial settings when improved sintered density and mechanical performance are needed. For comparison, the ASC 300 powder was compacted and sintered at the same temperature as the micro/nano bimodal powder compacts.

### Optical microscopy

The sintered compacts were mounted using hot mounting press. The mounts were then ground and polished using the standard metallographic procedure. Optical microscopy was performed on the duly prepared samples to evaluate the fraction of pearlite. The Trainable Weka Segmentation tool, part of the FIJI ImageJ freeware^25,26^, was used for this purpose.

### Scanning electron microscopy

High-resolution scanning electron microscopy (SEM) was performed using an LEO Gemini 1550 electron microscope (Carl Zeiss-LEO, equipped with a field emission gun: FEG-SEM) to evaluate the change in microstructure between the sintered compacts containing iron nanopowder and those containing carbon-coated iron nanopowder.

### Density

The green density of the compacts was evaluated using a micrometre and a simple balance with an accuracy of 0.0001 g. The micrometre was used to measure the height and diameter of the cylinder and calculate the volume of the compact. The density of the sintered compacts was measured using the Archimedes principle. Porosity was assessed from optical micrographs taken at different locations with the aid of the ImageJ, image analysis software and cross checked with the sintered density obtained from the Archimedes principle measurements. The porosity in the sintered compacts was analysed using the threshold function in the ImageJ software. The fractions of phases in a microstructure are calculated by determining the area that they occupy. The phase fraction obtained varied for different threshold values. It is essential to choose the optimum threshold value that shades only the porosity. The area fraction of the porosity is thus calculated.

### Hardness

Apparent hardness testing was performed using a Struers DuraScan 70G5 (Ballerup, Denmark) Vickers hardness tester at a 1 kg load on metallographic cross-sections of the sintered compacts.

### JMatPro

JMatPro 10.2 was in conjunction with a general steel database used to depict the phase fractions and phase transformations for the global alloy composition of the sintered materials under study.

## Supplementary Information

Supplementary Information.

## Data Availability

The datasets used and/or analysed during the current study available from the corresponding author on reasonable request.

## References

[1] Höganäs. Material and powder properties. in *Höganäs handbook for sintered components* 86 (2013).

[2] Oh JW, Seong Y, Shin DS, Park SJ (2019). Investigation and two-stage modeling of sintering behavior of nano/micro-bimodal powders. Powder Technol..

[3] Manchili SK, Wendel J, Hryha E, Nyborg L (2021). Sintering of bimodal micrometre/nanometre iron powder compacts—A master sintering curve approach. Powder Technol..

[4] Choi J-P, Lyu H-G, Lee W-S, Lee J-S (2014). Densification and microstructural development during sintering of powder injection molded Fe micro–nanopowder. Powder Technol..

[5] Edelstein, A. & Cammaratra, R. *Nanomaterials: Synthesis, Properties and Applications* (Institute of Physics Publishing, 1998).

[6] Asoro MA, Kovar D, Ferreira PJ (2014). Effect of surface carbon coating on sintering of silver nanoparticles: In situ TEM observations. Chem. Commun..

[7] Price SP, Henzie J, Odom TW (2007). Addressable, large-area nanoscale organic light-emitting diodes. Small.

[8] Crone B (2001). Electronic sensing of vapors with organic transistors. Appl. Phys. Lett..

[9] Kruis FE, Fissan H, Peled A (1998). Synthesis of nanoparticles in the gas phase for electronic, optical and magnetic applications—A review. J. Aerosol. Sci..

[10] Zhang H, Chen J, He Y, Xue X, Peng S (1998). The preparation of carbon-coated iron nanocrystals produced from Fe_2_O_3_-containmg composite anode in arc discharge. Mater. Chem. Phys..

[11] Qiu J (2004). Synthesis of carbon-encapsulated nickel nanocrystals by arc-discharge of coal-based carbons in water. Fuel.

[12] Bystrzejewski M, Huczko A, Lange H (2005). Arc plasma route to carbon-encapsulated magnetic nanoparticles for biomedical applications. Sens. Actuators B Chem..

[13] Sun G, Li X, Wang Q, Yan H (2010). Synthesis of carbon-coated iron nanoparticles by detonation technique. Mater. Res. Bull..

[14] Danninger, H. & Gierl-Mayer, C. 7—Advanced powder metallurgy steel alloys. in *Advances in Powder Metallurgy* (eds. Chang, I. & Zhao, Y.) 149–201 (Woodhead Publishing, 2013). 10.1533/9780857098900.2.149.

[15] Manchili SK (2020). Effect of nanopowder addition on the sintering of water-atomized iron powder. Metall. Mater. Trans. A..

[16] Díaz J, Paolicelli G, Ferrer S, Comin F (1996). Separation of the and components in the C1s photoemission spectra of amorphous carbon films. Phys. Rev. B: Condens. Matter Mater. Phys..

[17] Aarva A, Deringer VL, Sainio S, Laurila T, Caro MA (2019). Understanding X-ray spectroscopy of carbonaceous materials by combining experiments, density functional theory, and machine learning. Part I: Fingerprint spectra. Chem. Mater..

[18] Luo P, Nieh TG, Schwartz AJ, Lenk TJ (1995). Surface characterization of nanostructured metal and ceramic particles. Mater. Sci. Eng. A.

[19] Manchili SK (2018). Surface analysis of iron and steel nanopowder. Surf. Interface Anal..

[20] Manchili SK, Wendel J, Hryha E, Nyborg L (2020). Analysis of iron oxide reduction kinetics in the nanometric scale using hydrogen. Nanomaterials.

[21] Wendel J, Manchili SK, Cao Y, Hryha E, Nyborg L (2020). Evolution of surface chemistry during sintering of water-atomized iron and low-alloyed steel powder. Surf. Interface Anal..

[22] Wendel J, Manchili SK, Hryha E, Nyborg L (2020). Reduction of surface oxide layers on water-atomized iron and steel powder in hydrogen: Effect of alloying elements and initial powder state. Thermochim. Acta.

[23] Hryha E, Nyborg L, Alzati L (2015). Dissolution of carbon in Cr-prealloyed Pm steels: Effect of carbon source. Powder Metall..

[24] Sopinskyy MV (2014). Possibility of graphene growth by close space sublimation. Nanoscale Res. Lett..

[25] Arganda-Carreras I (2017). Trainable Weka segmentation: A machine learning tool for microscopy pixel classification. Bioinformatics.

[26] Schindelin J (2012). Fiji: an open-source platform for biological-image analysis. Nat. Methods.

