# Supplementary material for: Carbon-coated iron nanopowder as a sintering aid for water-atomized iron powder
Source: Sci Rep. 2022 Oct 25;12:17850. doi: 10.1038/s41598-022-22336-4 (PMC9596440; doi:10.1038/s41598-022-22336-4)
Supplement: Supplementary file 1 — Supplementary Information. [file 41598_2022_22336_MOESM1_ESM.pdf]

Carbon-coated iron nanopowder as a sintering aid for water-atomized iron powder

Swathi K. Manchili*, F. Liu, E. Hryha, L. Nyborg*
Department of Industrial and Materials Science, Chalmers University of Technology, Gothenburg, SE-41258, Sweden
*Corresponding author: swathi.manchili@gmail.com, lars.nyborg@chalmers.se

Supplementary Information

Figure S.1 shows the morphology that is so characteristic for carbon nanotubes that it is routinely used to count the number of layers in the carbon nanotube. On the other hand, the oxide layer on the Fe NP shows more complex lattice structure in HR TEM images (Figure S.2). The left inset shows the profile of integrated brightness vs distance inside the blue box. The measured values are 1.975 Å and 60°, which matches well. Additionally, the extracted Fourier transform pattern from the oxide region (red box) was indexed as [1-21-3] zone axis of Fe_2O_3.

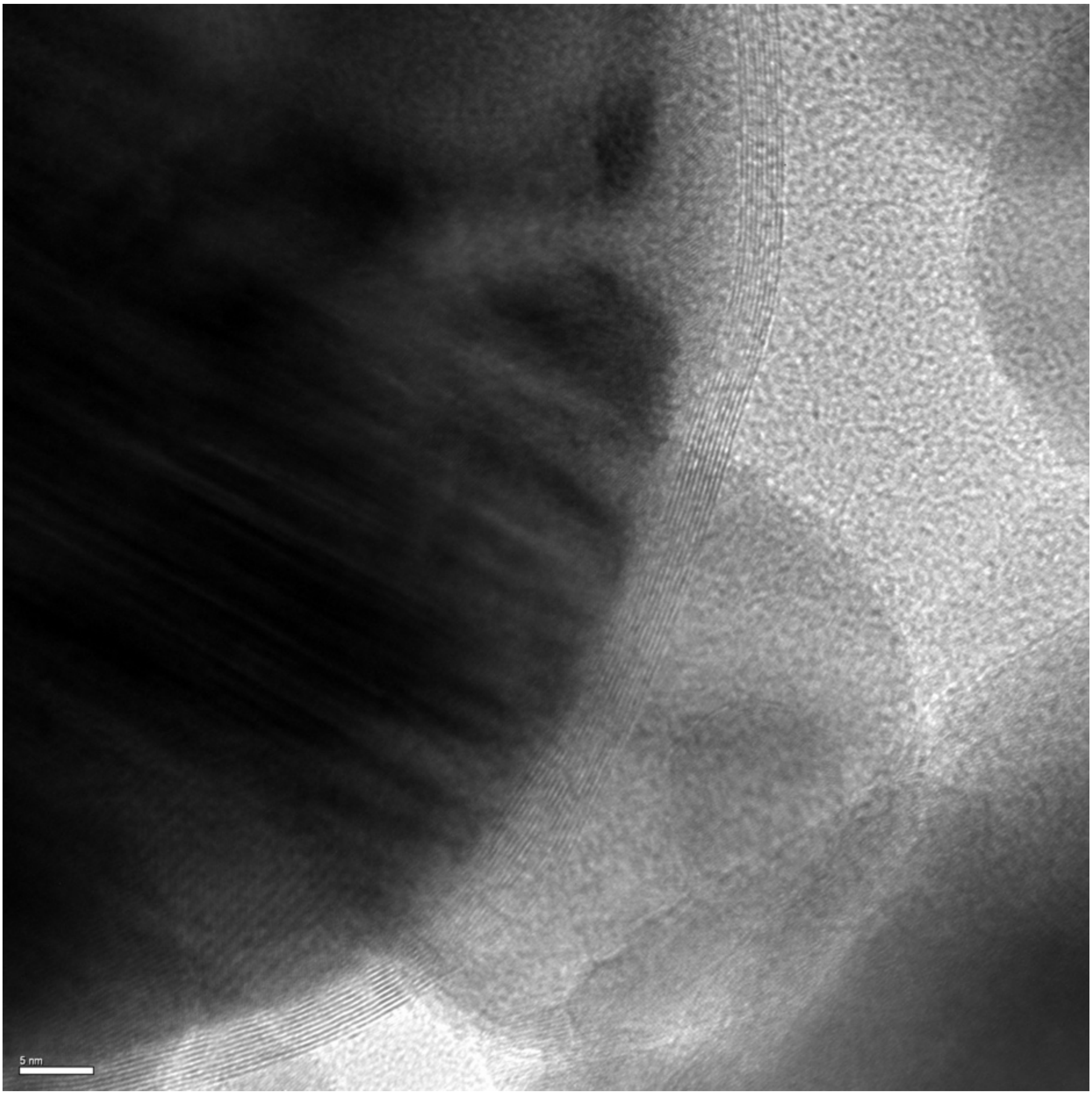

Figure S.1: HRTEM image of CC NP showing the graphitic layers.

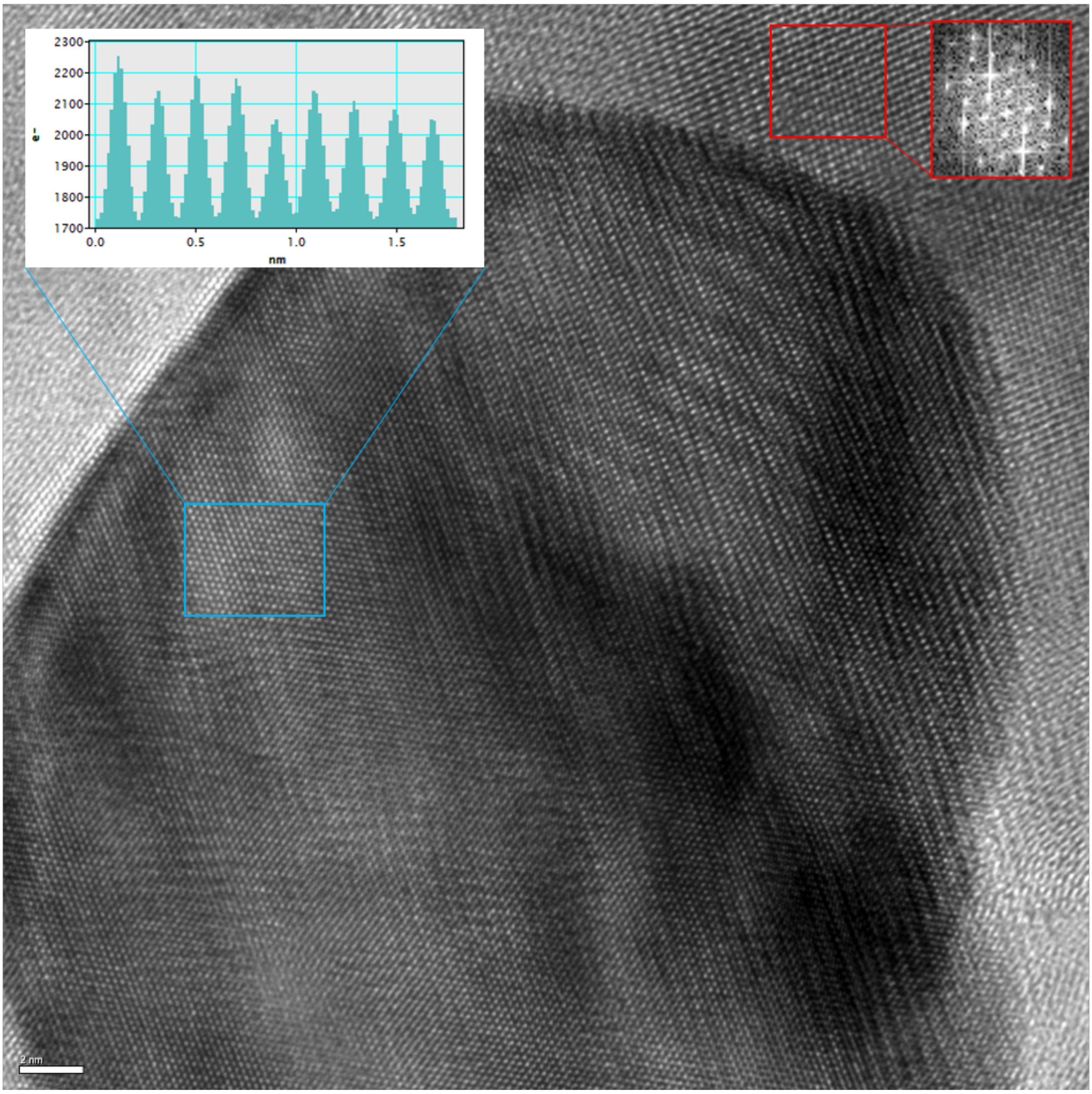

Figure S.2: HRTEM image of Fe NP showing the details of metallic iron core and oxide shell.

Figure S.3 is an attempt made using TEM images and ImageJ freeware to know the distribution of particle size. The graphs have been given below for the nanopowder variants. For CC NP, maximum particles were measured to be around 30 nm. A total of around 600 particles were analysed. For Fe NP, maximum particles were measured to be below 50 nm. A total of around 800 particles were analysed.

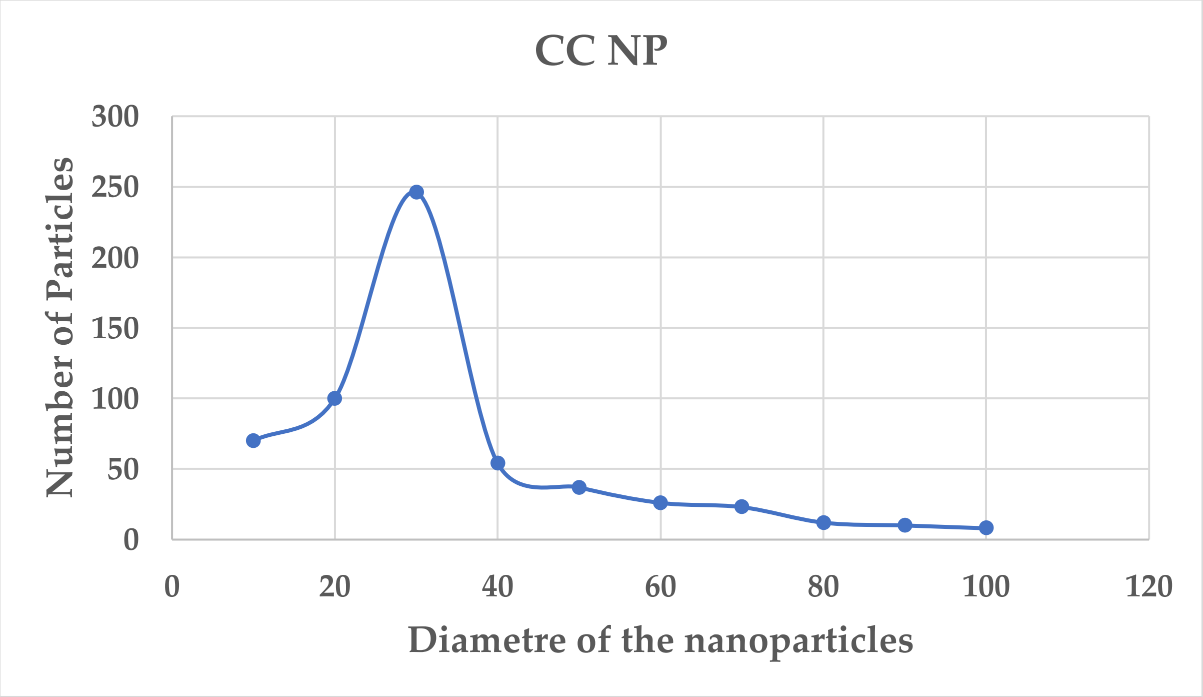

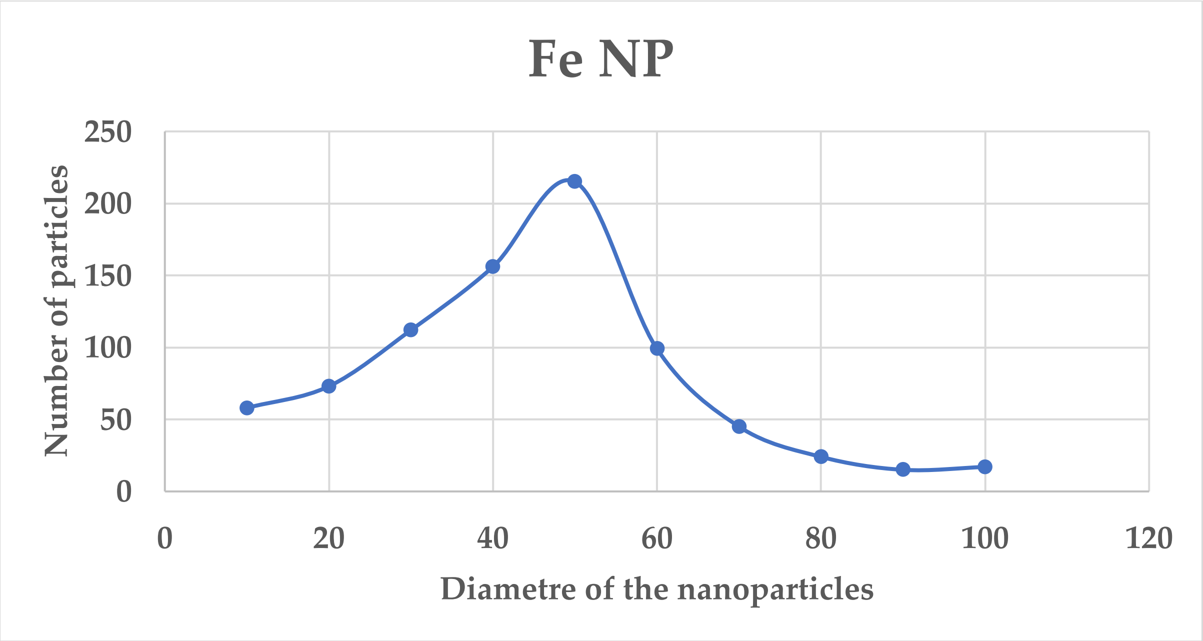

Figure S.3: Particle size distribution of CC NPs and Fe NPs.
